# Inheritance and Quantitative Trait Loci Mapping of Aromatic Compounds from Clementine (*Citrus* × *clementina* Hort. ex Tan.) and Sweet Orange (*C.* × *sinensis* (L.) Osb.) Fruit Essential Oils

**DOI:** 10.3390/genes14091800

**Published:** 2023-09-14

**Authors:** Vincent Ferrer, Gilles Costantino, Noémie Paymal, Carole Quinton, Estefania Carrillo Perdomo, Mathieu Paoli, Pierre Mournet, Patrick Ollitrault, Félix Tomi, François Luro

**Affiliations:** 1 UMR AGAP Institut, Université Montpellier, CIRAD, INRAE, Institut Agro, 20230 San Giuliano, France; vincent.ferrer@inrae.fr (V.F.); gilles.costantino@inrae.fr (G.C.); estefania.carrillo-perdomo@inrae.fr (E.C.P.); 2 Rémy Cointreau—Les Molières, 49124 Saint-Barthélemy-d’Anjou, France; noemie.paymal@remy-cointreau.com (N.P.); carole.quinton@remy-cointreau.com (C.Q.); 3 UMR SPE 6134—Université de Corse—CNRS, 20000 Ajaccio, France; paoli_m@univ-corse.fr (M.P.); tomi_f@univ-corse.fr (F.T.); 4 CIRAD, UMR AGAP Institut, Université Montpellier, CIRAD, INRAE, Institut Agro, 34398 Montpellier, France; pierre.mournet@cirad.fr

**Keywords:** blood sweet orange, essential oil yield, genotyping by sequencing, single-nucleotide polymorphism markers, genetic linkage map, quantitative trait loci

## Abstract

Despite their importance in food processing, perfumery and cosmetics, the inheritance of sweet orange aromatic compounds, as well as their yield in the fruit peel, has been little analyzed. In the present study, the segregation of aromatic compounds was studied in an F1 population of 77 hybrids resulting from crosses between clementine and blood sweet orange. Fruit-peel essential oils (PEOs) extracted by hydrodistillation were analyzed by gas chromatography coupled with flame ionization detection. Genotyping by sequencing was performed on the parents and the hybrids. The resulting “clementine × sweet blood orange” genetic map consists of 710 SNP markers distributed in nine linkage groups (LGs), representing the nine citrus chromosomes, and spanning 1054 centimorgans. Twenty quantitative trait loci (QTLs) were identified, explaining between 20.5 and 55.0% of the variance of the major aromatic compounds and PEO yield. The QTLs for monoterpenes and aliphatic aldehydes predominantly colocalized on LGs 5 and 8, as did the two QTLs for PEO yield. The sesquiterpene QTLs were located on LGs 1, 3, 6 and 8. The detection of major QTLs associated with the synthesis of aliphatic aldehydes, known for their strong aromatic properties, open the way for marker-assisted selection.

## 1. Introduction

With approximately 70 million tons, sweet orange (*Citrus* × *sinensis* (L.) Osb.) is the most widely produced citrus fruit in the world. The orange juice industry generates approximately 60,000 tons of peel essential oils (PEOs), which represent a major product for flavoring beverages food products, and cosmetics [1,2]. The main volatile compounds of the essential oils present in the oil glands of sweet orange peel are monoterpenes, followed by aliphatic compounds and sesquiterpenes [3]. Terpenes belong to one of the most diverse families of molecules in living organisms and have multiple functions [4]. There are over 30,000 different terpenes, and almost all originate from the same initial substrate called isopentenyl diphosphate (IPP) [5,6]. This initial substrate is composed of five carbons that branch successively to create precursors of monoterpenes (C10), sesquiterpenes (C15) or diterpenes (C20). Sesquiterpenes are synthesized primarily in the cytosol via the mevalonic acid pathway, whereas monoterpenes and diterpenes are synthesized mainly through the methylerythritol phosphate pathway (also known as the non-mevalonic pathway) in plastids [7]. Subsequently, the terpene synthases transform the precursors (C10, C15 and C20) into monoterpenes, sesquiterpenes and diterpenes. The diversity of terpenes is explained by the fact that there are many families of terpene synthases and by the fact that these terpene synthases can produce several different compounds [6,8]. The aliphatic compounds present in essential oils are synthesized from linoleic acid, which, through a series of reactions, produces aldehydes that can then be reduced to alcohols and transformed into esters [7].

Dozens of monoterpene synthases and sesquiterpene synthases have been sequenced and characterized from mRNAs in citrus [9,10,11,12,13,14,15]. The characterization of terpene synthases in citrus fruits and, in particular, of a limonene synthase allowed the study of its function by inactivating the enzyme by gene transformation. The reduction in limonene in the oil glands of fruit peel makes them less susceptible to attack by insects and microorganisms [16,17]. These transgenic organisms also provide insights into the role of a number of volatile compounds that characterize the aroma of orange juice. Indeed, significant variations in the levels of limonene (by more than 50%) and linalool (×3) in the juices do not seem to have an impact on aroma, whereas similar variations in esters (×3) clearly modify the aroma [18].

Essential oils are the basis of the aroma of sweet oranges (and citrus in a broad sense), and more information on their heritability and genetic control is still needed. The first sweet orange tree (*C.* × *sinensis*) was likely a unique genotype resulting from a spontaneous cross between wild mandarin (*Citrus reticulata* Blanco) and wild pummelos (*Citrus maxima* (Burm.) Merr.) [19]. Sweet orange has been spread over the centuries by vegetative propagation (initially by somatic embryony and since a few centuries ago by grafting), and its phenotypic diversification mainly relies on mutation selection [2]. Sweet orange was the first citrus species whose genome was sequenced [20]. Nevertheless, a year later, the International Citrus Genetics Consortium (https://citrusrdf.org/, accessed on 25 June 20232) published the sequence of the clementine (*Citrus* × *clementina* Hort. ex Tan.) genome [19]. The clementine genome is used by several international groups as a reference genome for citrus genomics studies as it derives from a haploid genotype that greatly facilitates sequence assembly. Clementine originated in Algeria in the late 19th century from a cross between a mandarin and a sweet orange tree [21]. Alquézar et al. [22] used the orange genome sequence to identify and locate approximately 50 terpene synthases responsible for the emission of major terpenes. Phylogenomic analysis of these terpene synthases revealed five genetic clusters. The authors also studied the expression of approximately ten sesquiterpene synthases.

Numerous crosses have been made between citrus species, mainly of the mandarin–pummelo complex, to create varieties with new aromatic profiles and to study the inheritance and variability of aromatic compounds. For example, the aromatic compounds in citrus fruit juice have been investigated in mandarins and their hybrids, such as tangors and tangelos, which originate from a cross between a mandarin and a sweet orange and a mandarin and a grapefruit (*Citrus* × *paradisi* Macf.), respectively [23,24,25]. The authors showed qualitative and quantitative variations, with mandarins characterized by fewer volatiles and more aldehydes and hybrids with sweet orange having more sesquiterpenes and esters. However, there are very few works actually describing the aroma of progenies. Miyazaki et al. [25] identified 22 compounds by GC-O, whose olfactory intensities varied between hybrids. These compositional variations impacted the overall aromatic profile of the hybrids with orange, grapefruit, tangerine (spicy, woody) and even pumpkin fragrances for a number of genotypes.

The composition of the essential oil of citrus fruit peels in hybrid populations has also been studied [26,27,28,29,30]. A diversity of compositions has been observed for most essential oils, even though limonene still represents more than 80% of the total aromatic compounds. However, these studies did not investigate the aromatic properties by sensorial analysis of the different PEO compositions. Tomi et al. [31] showed that most of the major leaf aromatic compounds segregated between hybrids of a “clementine × mandarin” progeny. The observed segregation for major compounds suggests that essential oil production could also have polygenic control. In citrus, the genetic architecture of internal and external quality traits of mandarin fruits is being increasingly studied [32,33,34,35].

However, the inheritance of citrus aromatic compounds needs to be more deeply studied. Yu et al. [36] conducted a study on the heritability of volatile compounds in citrus juice and detected 206 QTLs for 94 volatile compounds using an Illumina GoldenGate 1536-SNP microarray [36]. Of these 206 QTLs, 25 remained stable over two years of sampling. The authors observed that many of these QTLs colocalized, particularly for compounds of the same metabolic pathway. Another study addressed the role of linalool in the response of citrus leaves to citrus canker (*Xanthomonas citri* subsp. *citri*) [37]. The authors observed colocalization between the QTL for canker resistance and that for linalool content. They suggested that a high linalool content is one of the factors explaining the resistance of some varieties to canker.

The aim of the work presented here is two-fold: (i) to study the inheritance of PEO production and aroma compounds in a segregating population originating from the “clementine/orange” cross and detect QTLs controlling the production of PEOs and (ii) to decipher the complexity of the sensory profile of blood sweet orange and clementine using the variability of the segregating population.

## 2. Materials and Methods

### 2.1. Biological Material

The F1 population consisted of 92 hybrids from two crosses: clementine (SRA 63) (*C.* × *clementina*) × Sokotoro sweet orange (SRA 407) (*Citrus* × *sinensis*) and clementine (SRA 63) × Moro sweet orange (SRA 301). The two populations can be considered one because the two sweet orange cultivars originated from the same “bloody pulp” group, and their PEOs did not differ in composition or quality [2]. Sweet oranges evolved only by mutations modifying very few phenotypic characters, such as pulp coloration due to anthocyanin synthesis [38].

All trees of parents and progenies were planted in 1998, grouped in the same orchard, grafted on the same rootstock of Carrizo citrange and grown under the same conditions [39]. The fruits of 77 hybrids were harvested in the same week of December 2020 in sufficient quantity to obtain approximately 200 g of fresh peel from which a sufficient volume of essential oils was extracted for the subsequent analysis. The collected fruits were weighed, and the zest (flavedo) was removed using a knife previously disinfected with 70% ethanol. The fresh zests were weighed and immediately stored in the freezer at −20 °C until subsequent analysis.

For 77 hybrids, the zest of one fruit was weighed to determine the percentage of dry mass of the zest. For this purpose, fresh peels were weighed and then oven-dried at 45 °C for 48 h until the dry mass stabilized.

### 2.2. Essential Oil Extraction

The frozen zest was mixed with distilled water and then ground using a 1300 W blender (Magimix^®^, Vincennes, France) for one minute. All samples were distilled in a 2 L flask with 1 L of distilled water. Using an EM2000/CE flask heater (Electrothermal^®^, London, UK), the mixture was boiled for 2.5 h. The essential oil was collected via a Clevenger cooled to 4 °C by a glycol/water mixture and set in motion by a model C20 circulating thermostat (Huber^®^, Offenburg, Germany). The collected PEO was weighed, and 300 µL of PEO was stored in stained glass tubes filled to the maximum to avoid the presence of air. The remaining PEO was used for sensory analysis. The tubes containing essential oils were stored at −20 °C.

### 2.3. Chemical Composition Analysis

Gas chromatography (GC) analysis was performed using a Clarus 500 (Perkin Elmer, Courtaboeuf, France) chromatograph equipped with a splitter injector, two capillary columns (50 m × 0.22 mm i.d.; film thickness: 0.25 μm), namely a polar (BP-20, polyethylene glycol) and an apolar (BP-1, polymethylsiloxane) column, and flame ionization detectors. The operating conditions were as follows: the carrier gas was hydrogen, the column head pressure was 20 psi, the flow rate was 1 mL/min, the injector temperature was 250 °C, and the detector temperature was 250 °C. The temperature programming was from 60 to 220 °C at 2 °C/min, followed by a 20 min step at 220 °C. The injection was performed in split mode at a ratio of 1/60.

The compound proportions of the oils were expressed in g per 100 g from the chromatogram peak area using the response factors for each class of compounds [40]. The compound used as the internal reference grade was nonane, and each oil sample was prepared using the following volumetric proportions: 1.00/11.75/487.25 (nonane/oil/chloroform) (Sigma–Aldrich, St. Louis, MO, USA).

Gas chromatography–mass spectrometry (GC–MS) analyses were performed using a Perkin Elmer Auto System XL chromatograph with two automatic injectors and two capillary columns (50 m × 0.22 mm i.d.; film thickness: 0.25 μm), i.e., the polar (BP-20, polyethylene glycol) and apolar (BP-1, polymethylsiloxane) column, coupled with a Perkin Turbo Mass detector. The molecules were bombarded in an ionization source by an electron beam of 70 eV. The detection was performed by a quadrupole analyzer made of an assembly of four parallel electrodes of a cylindrical section. The carrier gas was helium, and the pressure at the head of the column was 43 psi. The flow rate was 0.8 mL/min. The program used included a temperature rise from 60 to 220 °C by 2 °C per minute with a plateau at 220 °C for 20 min. The injection was performed in divider mode with a division ratio of 1/75.

Two methods were used to identify the compounds: (1) by comparison of the retention indices on apolar and polar columns, calculated from the retention times of a series of alkanes (by linearly interpolating these times from the times of the pure compounds and from the literature data) and (2) by comparing their mass spectra with the spectra from the National Institute of Standards and Technology (NIST) database.

The essential oils of seventy-seven hybrids, as well as those of the three parents, namely clementine and the two orange cultivars, were analyzed by GC. The same quantity of nonane was added to each PEO sample and used as the internal standard for quantitative evaluation of each compound expressed in g/100 g of PEO, equivalent to a percentage.

### 2.4. Sensorial Analysis

Two experts in sensorial analysis analyzed the PEO, indicating whether the sample had an orange or clementine zest aroma note. An intensity scale ranging from 0 to 4 was used, with 0 indicating no similarity and 4 indicating the maximum intensity of the “orange” or “clementine” aroma note.

### 2.5. Statistical Analysis

The diversity of the progeny and their parents was represented via a heatmap using the basic package of RStudio software. The relative quantity values of each of the 31 aromatic compounds were centered and reduced prior to visualization.

### 2.6. GBS and Marker Filtering

The protocol was identical to that used by Oueslati et al. [41]. DNA from 92 hybrids and parents (Moro, Sokotoro and 2 clementines) was extracted on the AGAP Institut genotyping platform in Montpellier using the DNeasy^®^ Kit (Qiagen, Hilden, Germany) following the manufacturer’s instructions. Genomic DNA concentrations were then adjusted to 20 ng/μL. The library was made using the restriction enzyme *Ape*KI (New England Biolabs, Hitchin, UK). Ten microliters of each DNA sample (200 ng) was digested with the restriction enzyme. The whole set was incubated at 75 °C for 2 h and then 65 °C for 20 min to inactivate the enzyme. The ligation reaction was performed in the same plate with T4 DNA ligase (New England Biolabs, Hitchin, UK) at 22 °C for 1 h. Finally, the ligase was inactivated before pooling the samples in the same tube, and the DNA was amplified by PCR. To reduce genome complexity, primers with a selective base (A) were used to perform the amplification [42]. The amplified DNAs were then sequenced by Genewiz (Leipzig, Germany) in “paired end” mode (150 bp × 2) using an Illumina HiSeq4000 sequencer. Genewiz provided Fastq files.

Adapters were removed with fastp v.0.21.0 software (https://github.com/OpenGene/fastp, accessed on 8 December 2020). Variant calling was performed using the clementine genome v1.0 as a reference (https://phytozome-next.jgi.doe.gov/info/Cclementina_v1_0/, accessed on 8 December 2020). The VcfHunter v1.2.0 program (https://github.com/SouthGreenPlatform/VcfHunter/, accessed on 9 December 2020) was used to perform variant calling. The parents were genotyped twice to facilitate and give robustness to the classification of marker segregation in the different hybrids of the crosses.

### 2.7. Construction of the Consensus Genetic Map

Only polymorphic markers were kept, i.e., markers heterozygous in one parent and homozygous for the other parent or heterozygous in both parents (‘0/0 × 0/1’, ‘0/1 × 0/0’, ‘0/1 × 1/1’, ‘1/1 × 0/1’ and ‘0/1 × 0/1’). A chi-squared test of goodness of fit (*p* ≥ 0.05) was performed on all markers. For markers with a 1:1 segregation ratio (‘0/0 × 0/1’, ‘0/1 × 0/0’, ‘0/1 × 1/1’ and ‘1/1 × 0/1’), 3.841 was set as the threshold for deviation from expected Mendelian segregation. For markers with a 1:2:1 ratio (‘0/1 × 0/1’), the threshold was 5.991. Genotypes suspected of resulting from sequencing errors were transformed into missing data. Markers fewer than 500 base pairs away from adjacent markers and with identical segregation were removed. Individuals with more than 20% missing data were excluded, as were markers with more than 10% missing data.

The genetic map was created using JoinMap^®^5 software (https://www.kyazma.nl/index.php/JoinMap/, accessed on 25 June 2023). In the case of ‘0/1 × 0/0’ crosses, the progeny were coded ‘ll’ for ‘0/0’ and ‘lm’ for ‘0/1’. For the crosses ‘0/1 × 1/1’, the progeny were coded ‘ll’ for ‘1/1’ and ‘lm’ for ‘0/1’. For the cross ‘0/0 × 0/1’, the progeny were coded ‘nn’ for ‘0/0’ and ‘np’ for ‘0/1’. For the cross ‘1/1 × 0/1’, the progeny were coded ‘np’ for ‘0/1’ and ‘nn’ for ‘1/1’. Finally, for the cross ‘0/1 × 0/1’, the progeny were coded ‘hh’ for ‘0/0’, ‘hk’ for ‘0/1’ and ‘kk’ for ‘1/1’. The type of cross selected when importing the data was cross-pollinated (CP). The ‘population node’ function was used, and 9 linkage groups (LGs) were formed based on an independence log of the odds (LOD) greater than or equal to 4. These 9 LGs corresponded to the citrus basic chromosomes. The genetic map was constructed using the “regression mapping” method and the Haldane function for calculating the genetic distances between markers.

### 2.8. QTL Detection

The genetic map of the progeny was imported directly into MapQTL^®^6 software (https://www.kyazma.nl/index.php/MapQTL/, accessed on 11 January 2021) to identify the genomic regions involved in the synthesis of 31 volatile compounds, the yield of PEO and the aromatic characteristic notes of clementine and sweet orange. The cross-pollinated (CP) population type and the “interval mapping” method were used for quantitative trait locus (QTL) mapping. The significance threshold of the QTLs was calculated via a 1000-permutation test. Other calculation parameters were set to the MapQTL default. The threshold was set (*p* ≤ 0.05) for each linkage group and each phenotypic variable. Finally, the Kruskal–Wallis test was conducted independently on each locus for each phenotypic variable. The LOD score for each parameter and linkage group was calculated using the “qqman” package in R [43]. LOD peaks were used to determine the position of significant QTLs on chromosomes. The total variance explained by the QTLs was calculated with the equation % Var Expl. QTL = 100 × (H_0_var_ − var)/Var_pop_, in which H_0_var_ = residual variance under the null hypothesis, var = residual variance after fitting the QTL and Var_pop_ = population variance.

The GFF database (https://phytozome-next.jgi.doe.gov/info/Cclementina_v1_0/, accessed on 17 July 2023) was used to identify potential candidate genes linked to the QTLs. For gene functional annotation, the following databases were used: g:Profiler version e109_eg56_p17_1d3191d (https://biit.cs.ut.ee/gprofiler/gost/, accessed on 18 July 2023) and Citrus clementina Ensembl (https://plants.ensembl.org/Citrus_clementina/Info/Index/, accessed on 18 July 2023). To complete the gene-identification process, a search for enzymes involved in terpenoid metabolism was also carried out using the KEGG database (https://www.kegg.jp/pathway/cic00900, accessed on 18 July 2023), specifying the steps in the biochemical process of terpenoid biosynthesis.

## 3. Results

### 3.1. Essential Oil Yield of the Segregating Population

**Figure 1:**
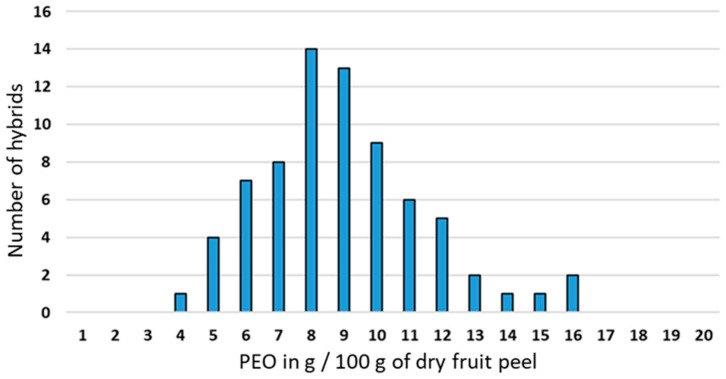
Histogram depicting the distribution of essential oil yield expressed in g for 100 g of dry mass of peel of the population of 77 hybrids and the 2 parents.

Essential oil yields per 100 g of dry peel (DP) vary greatly, and the distribution of the number of hybrids by yield class appears to follow a Gaussian (normal) distribution with a mean value of 8.3 g/100 g DP and minimum and maximum values of 5.0 and 15.3 g/100 g DP, respectively (Figure 1 and Supplementary Materials Table S1). Clementine and orange have yields of 6.0 and 9.7 g/100 g DP, respectively. Although the majority of individuals (44/77) show performance values between the values of the two parents, a high proportion (43 g/100 g DP) shows transgressive segregation.

### 3.2. Composition, Segregation and Variation of PEO Compounds in the Progeny and Their Parents

Seventy-seven compounds were identified among the seventy-seven individuals of the population (Supplementary Materials Table S1). Considering the main class of aromatic compounds, all individuals have compositions globally similar to those of the parents, i.e., dominated by monoterpenes representing between 97.18 and 99.96% of the total composition. Aliphatic compounds vary in representation between 0 and 2.3%, and sesquiterpenes vary between 0 and 1.7%. Traces of unknown compounds and diterpenes were also detected. The two sweet orange parents of the population have very similar profiles. Five minor compounds (<0.1 g/100 g%) are present in only one of the two oranges: trans sabinene hydrate (Sokotoro) and terpinolene p-mentha-1,8-dien-4-ol, 10-limonenyl acetate and valencene (Moro). Four compounds are present in only one analysis of the two clementine PEOs: -(2E,6Z)-dodecadienal, perillyl acetate, α-thujene and 2-hexenal.

We identified 30 compounds that are present only in the progeny and therefore absent from both parents (number of hybrids where they are present): hexanal (6); p-cymene (6); (Z)-β-ocimene (1); β-thujone (2); cis-p-menth-2-en-1-ol (2); cis-limonene-1,2-epoxide (17); trans-pino carveol (1); nonan-1-ol, isogeranial (4); dihydro carvone (10); trans-piperitol (4); cis-p-menth-1-en-3-ol (1); octyl acetate (16); cis carveol (7); geraniol (1); linalyl acetate (1); isopiperitenone (9); perillaldehyde L (20); decanol (5); limonene-10-ol (13); trans carvyl acetate (2); α-terpinyl acetate (12); citronellyl acetate (4); neryl acetate (9); geranyl acetate (3); α-copaene (19); decyl acetate (5); β-caryophyllene (23); β-copaene (11); germacrene-D (8); and (E)-phytol (2).

**Figure 2:**
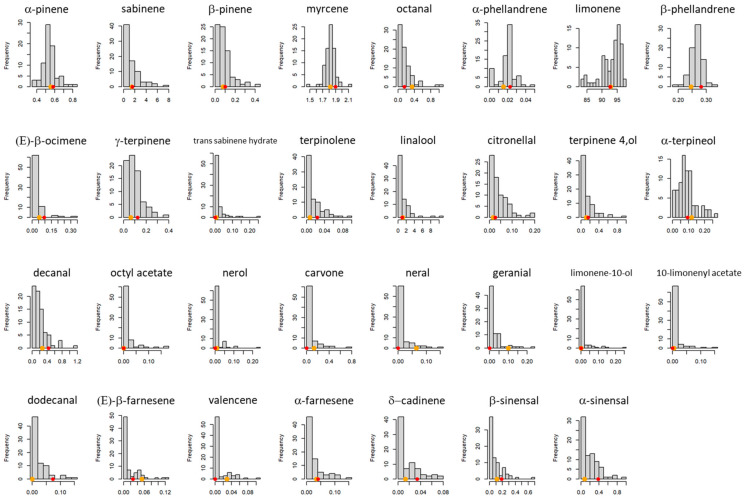
Distribution of the representation of 31 aromatic compounds (expressed in %(*w*/*w*)) in the population of clementine × orange hybrids; the clementine and orange values are represented by orange and red dots, respectively.

Thirty-one aromatic compounds were selected for further study based on their presence in at least one of the two parents, in the progeny and in the two sweet orange cultivars and clementine replicates. In the progeny, the amount of some aromatic compounds varied greatly between genotypes: 83.22 to 97.14% for limonene and 0.09 to 7.60% for sabinene. Among the oxygenated compounds, the representation of linalool varies between 0.04 and 10.63%. Among the aliphatic aldehydes, the representation of octanal varies between 0 and 1.10% and that of decanal varies between 0 and 17%. Among the sesquiterpenes, the representation of α-sinensal varies between 0 and 0.91%, and that of β-sinensal varies between 0 and 0.68%. A Gaussian distribution is not observed for all compounds (Figure 2). Only four compounds have a normal or approximately normal distribution: α-pinene, myrcene, α-phellandrene and β-phellandrene.

**Figure 3:**
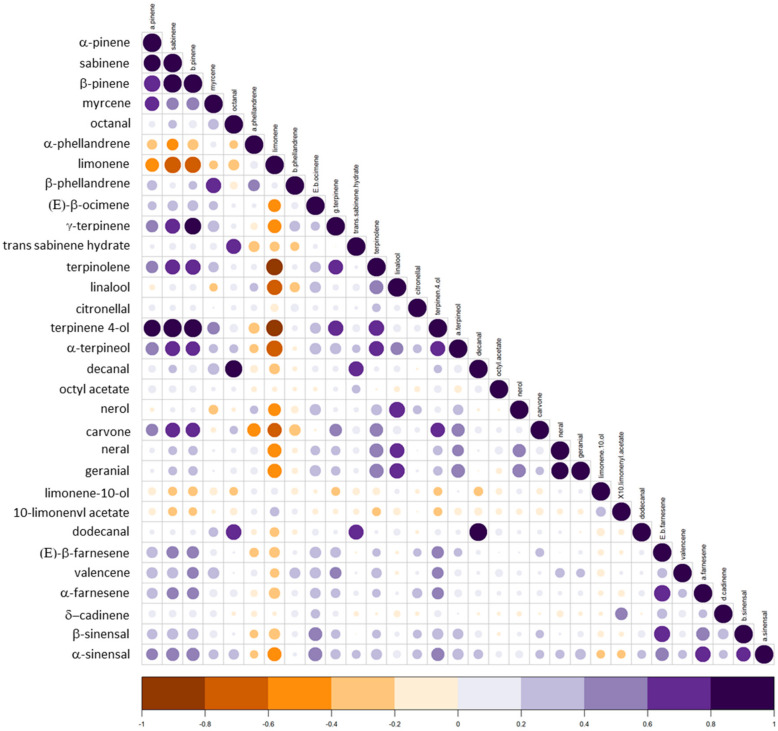
Correlations between the 31 major compounds. The colored disc indicates the strength of the correlation (coefficient).

Some compounds have highly positively correlated variations (coefficient > 0.8), such as terpinene-4-ol with α-pinene, sabinene and β-pinene; decanal is correlated with octanal and dodecanal; γ-terpinene is correlated with β-pinene; and finally, geranial is correlated with neral (Figure 3). The ultra-majority compound limonene has either negative or no correlations with the other compounds. The strongest negative correlations (coefficient <−0.8) with limonene are observed for terpinolene and terpinen-4-ol. Weaker negative correlations (coefficient between −0.8 and −0.6) exist between limonene and four compounds (sabinene, β-pinene, linalool and α-terpineol).

**Figure 4:**
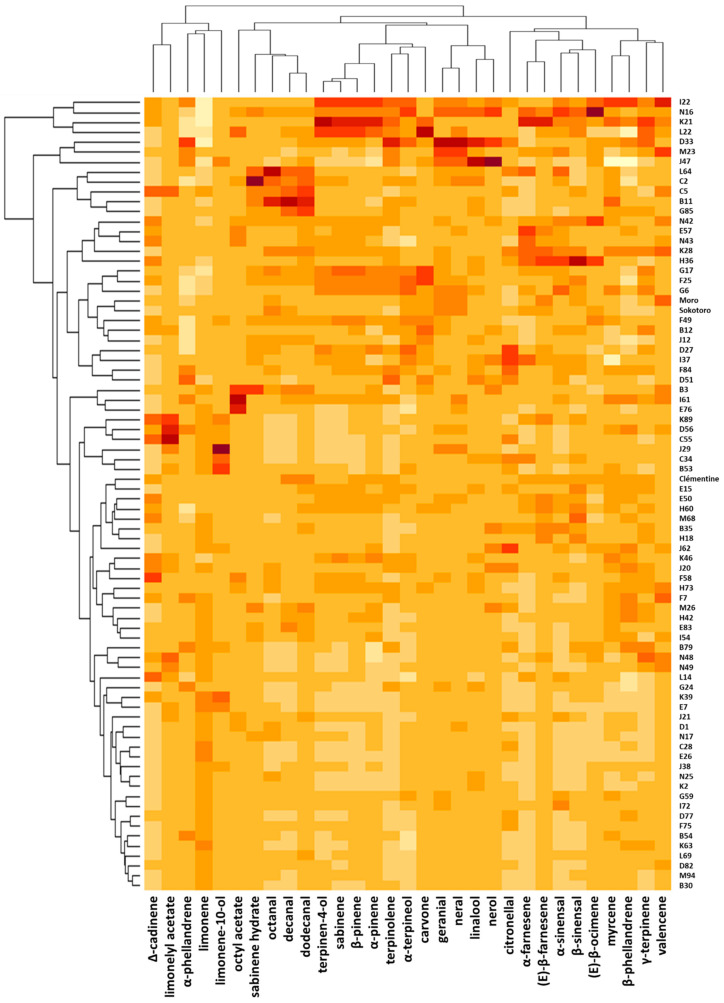
Heatmap representing the diversity of the 77 hybrids and their parents based on the amounts of the 31 major compounds. For each compound, the darker the box, the higher its value.

The diversity of the progeny of clementine × orange hybrids does not show any particular structure based on the 31 main aromatic compounds, even if the first 12 hybrids in the upper part of the heatmap seem to have very different profiles from the others (Figure 4 and Supplementary Materials Figure S1). Eighteen hybrids are distinguished from the group of individuals whose chemical profile is rather similar to those of the parents by their unique position in the PCA plot or their compound contents higher or lower than the population average on the heatmap. The main contributors to this dispersion of hybrid profiles in the PCA are limonene, decanal, dodecanal, octanal, sabinene, terpinene-4-ol and β-pinene. For example, hybrids D33 and J47 have a high linalool content (8.9 and 10.6%, respectively), while the average for the whole population is 1.2%, as for the two parents. The individual K21 stands out as having the highest proportion of sabinene (7.6%), while it is 1.5% in the parents and 1.4% on average for all of the hybrids. In these three hybrids, the proportion of limonene is the lowest in the whole population, between 83.4 and 84.9%.

### 3.3. Sensorial Profile of the Hybrids

**Figure 5:**
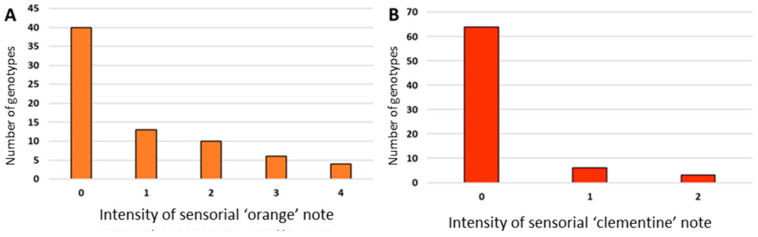
Histogram representing the distribution of the intensity of the sensory aroma notes (**A**) ‘orange’ and (**B**) ‘clementine’ among the progeny of 73 hybrids, from no aroma note (0) to very intense (4).

Sensorial analysis of PEO was performed by noting the presence or absence and the intensity of the aroma notes of sweet orange and clementine. An aromatic note of one of the two parental types does not mean that the corresponding PEO has an identical odor to one of the parents, simply that the sensorial expert detected it. Among the 77 PEOs chemically analyzed, the sensorial expert analyzed only 73. Within the hybrid population, the intensity of the ‘orange’ and ‘clementine’ sensorial notes of the hybrids was variable (Figure 5A,B). The ‘orange’ aroma note was more common in the progeny than the ‘clementine’ aroma note (33 vs. 7). Ten hybrids were characterized by a high intensity of the sweet orange aroma note (2 or more). The PEO of 33 hybrids had no aromatic parental aroma notes. No relationship was detected between the chemical composition of PEOs and the intensity of the ‘orange’ and ‘clementine’ aroma notes.

### 3.4. Genetic Map

A total of 18,940 SNPs were obtained by GBS, and among them, 2240 were polymorphic SNPs. After removing adjacent markers with identical segregation and markers with more than 10% missing data, as well as individuals with more than 20% missing data, 777 markers and 81 hybrids remained available for genetic mapping. The final consensus genetic map is composed of 710 SNP markers distributed in nine linkage groups for a total distance of 1054 cM (Table 1 and Supplementary Materials Table S2). Each linkage group was numbered according to the clementine reference map corresponding to the *C. clementina* V1.0 reference genome [19]. LG sizes ranged from 62 cM (LG6) to 187 cM (LG2) with an average density ranging from 1 SNP/4 cM (LG9) to 1 SNP/1 cM (LG3, LG5, LG6).

**Figure 6:**
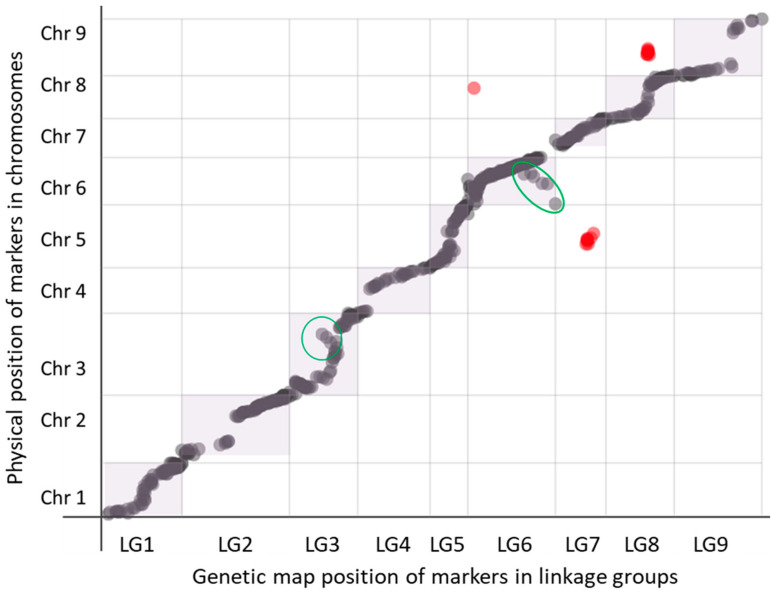
Marey diagram of the relationships between marker positions in the clementine pseudochromosome assembly Chr1 to Chr9 (ordinate) and positions on the genetic maps of LG1 to LG9 (abscissa). Red dots are SNP markers with congruent positions between the physical map and linkage groups. The green circle shows the zone with markers in the inverted position between the physical map and linkage group.

Comparison of the distances between markers on the genetic map and on the physical assembly of *C. clementina* V1.0 gives a sigmoidal representation of the LGs (visible particularly in LGs 1, 3, 5, 8 and 9) with areas in which the physical distance increases while the genetic distance plateaus (Figure 6). In general, synteny and collinearity are observed between the genetic and physical maps, except in a few small areas: three batches of markers (in red) positioned differently on the genetic map and on the physical map (6/8; 7/5 and 8/9) and two areas of inversion on linkage groups 3 and 6.

### 3.5. Inheritance of Volatile Compounds and Oil Yield

**Figure 7:**
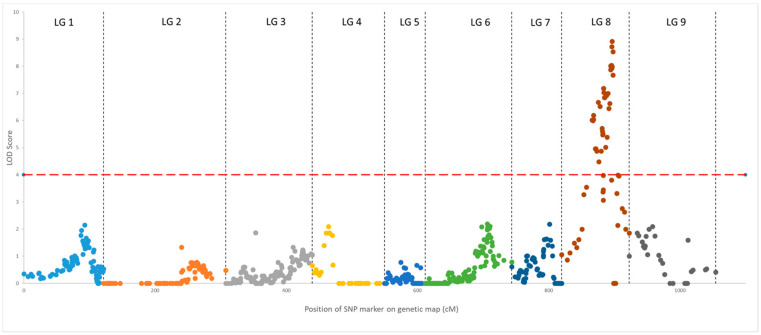
Manhattan plot representing SNP markers linked to decanal expression. Each point represents the genetic map position (in cM) of an SNP marker on an LG (abscissa) and its LOD score (on the ordinate) of linkage disequilibrium with decanal variation. The genetic distances on the *x*-axis are the sum of the lengths (in cM) of the nine linkage groups. The significance level (in red) of the LOD score was calculated via permutation test (*p* ≤ 0.05).

**Figure 8:**
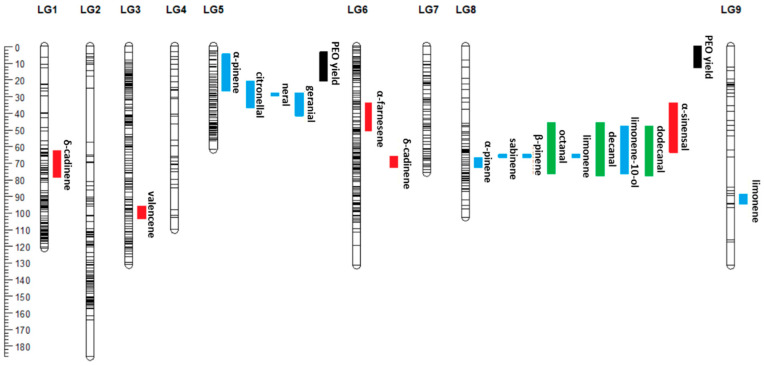
Graphical representation of the positions of QTLs on the 9 linkage groups with their marker density (horizontal black lines). The confidence intervals of the QTLs are represented to the right of each linkage group by colored rectangles: green corresponds to the aliphatic aldehydes, blue corresponds to the monoterpenes, red corresponds to the sesquiterpenes and black corresponds to PEO yield.

Markers related to quantitative variation in each of the compounds and yield are above the minimum significance level, as represented in the Manhattan plot (Figure 7 and Supplementary Materials Table S2). A total of 20 QTLs were identified in the clementine × orange hybrid population (Table 2). These QTLs explain a significant part of the variance in the traits (between 20.5 and 55.0%). Of these QTLs, eighteen control the expression of the different volatile compounds, and two control the global yield of essential oils in the peel. QTLs for compounds in the same chemical families generally colocalize. This is the case for sabinene, β-pinene, limonene-10-ol and limonene, with a QTL localized to linkage group 8 (Figure 8). In linkage group 8, the same QTL seems to control the following aliphatic aldehydes: octanal, decanal and dodecanal (Figure 8). The synthesis of neral, geranial, citronellal and α-pinene is controlled by several QTLs located in linkage group 5. QTLs associated with sesquiterpenes are distributed in linkage groups 1, 3, 4 and 8. Finally, two QTLs explaining the oil yield trait of the peel are located at the beginning of linkage groups 5 and 8. These two QTLs together explain 49.2% of the total variance in PEO yield (Table 2).

### 3.6. Research on the QTLs and Genes Associated with Terpenoid Biosynthesis

A total of 3475 genes and 2324 proteins were identified in the genomic regions of the *C. clementina* V1.0 [19] assembly corresponding to the 20 QTLs. An additional QTL for α-sinensal was prospected on scaffold 9 that corresponds to some SNP markers located in LG8 of the genetic map but belong to scaffold 9 in the reference genome (red-colored SNP markers in Figure 6). The number of genes and proteins depends on the size of the QTL, ranging from 24 genes for the neral QTL (scaffold 5) to 1075 genes for the dodecanal QTL (scaffold 8) (Supplementary File). Among the 13 QTLs for aromatic PEO compounds, 20 proteins or enzymes involved in terpene biosynthesis were identified (Table 3), including enzymes upstream of the monoterpene and sesquiterpene biosynthesis pathway: phosphomevalonate kinase [EC:2.7.4. 2] of the mevalonate pathway; 4-hydroxy-3-methylbut-2-enyl diphosphate reductase [EC:1.17.7.4] of the MEP/DOXP pathway; and geranylgeranyl pyrophosphate synthase [EC:2.5.1.1]. Geranylgeranyl pyrophosphate synthase is encoded by three genes close in the genome in the α-pinene and citronellal QTLs (scaffold 5). Twelve proteins and enzymes were identified downstream of the terpenoid biosynthesis pathway, including six for terpenoid–quinone biosynthesis.

## 4. Discussion

The clementine–sweet orange cross has created a diversity of aromatic profiles. However, this diversity is relatively low because the majority of individuals have chemical profiles that are fairly similar to each other and to those of the two parents. Other authors studying the same type of crosses have observed this low diversity [26,30]. This could be explained by the fact that orange and clementine are genetically related, as clementine is derived from a ‘Willowleaf’ mandarin × sweet orange cross [19]. Since sweet orange is a mandarin/pummelo interspecific hybrid and therefore highly heterozygous, one would expect greater compositional variation as the genetic diversity and chemical compositional diversity of the essential oil between mandarin and pummelo trees are very large [44]. The explanation for the low variation observed would then lie in the high limonene content in both parents (nearly 90%), which partially masks the variation in minor compounds. Citrus fruits belonging to other species, such as citron, lemon and some mandarins, have lower limonene contents [45,46,47]. However, in the clementine × orange progeny, some individuals are clearly different, with relatively low levels of limonene and high levels of linalool, sabinene, neral and geranial. These compounds present in significant amounts have been identified as markers of fruit immaturity in orange [48]. It is therefore possible that the fruits of these genotypes mature later than those of other hybrids from this cross. This type of transgressive phenotype has already been detected in progeny from crosses between closely related parental genotypes, such as orange/clementine [27] and clementine/mandarin [31].

Many compounds (**30**) are absent in the parents and present only in the offspring. It is likely that the majority of these compounds are at or below the limit of detection of GC and therefore not detected in the parents. Metabolic pathway complementation in the hybrids may also lead to the production of new compounds. It is also possible that hybridization results in rearrangements or epigenetic modifications of DNA that would activate or deactivate biosynthetic pathways [49]. Even if the compositional variation observed is small compared to that in the parents, we observed significant variation in the intensity of the ‘orange’ aroma note within the population, with ten individuals showing a strong ‘orange’ aroma note (>2). An ‘orange’ aroma note does not necessarily mean an aroma similar to that of orange; this character is detected but could be common. Moreover, no hybrid presents an aroma identical to that of orange. This type of profile is not surprising because hybrids with orange-like juice aroma profiles have already been identified and characterized in mandarin hybrids [50]. The ‘clementine’ aroma note is present in fewer hybrids and is on average of lower intensity than the ‘orange’ note. Unfortunately, no correlation between aroma descriptors (or notes) and compounds emerged in our study. This is likely because the ‘orange’ or ‘clementine’ aroma profile results from interactions between several compounds, some of which are present in very small amounts [51]. The relatively small number of hybrids in the progeny would also limit the possibility of obtaining a greater number of combinations between compounds and thus aroma profiles to achieve the combination, reproducing the parental aroma. The detection threshold of GPC is also an obstacle to the detection of minor compounds with a strong aromatic impact.

QTLs for volatile compounds belonging to the same chemical families generally colocalize, indicating that they likely control the same metabolic pathways [36]. Yu et al. [36] proposed two hypotheses to explain this phenomenon: the presence of several nearby loci on the chromosomes or a single locus with a pleiotropic effect. Alquezar et al. [22] suggest that terpene synthases organize into clusters in the genome that, following duplications, would undergo paralogous evolution, which would explain the colocalization of QTLs for several metabolites or the identification of several QTLs for the same molecule. It is also known that terpene synthases can produce different compounds in varying proportions [6]. In our study, the QTLs for aliphatic aldehydes and monoterpenes colocalized to linkage group 8, and some of the QTLs of monoterpenes colocalized to linkage group 5. This observation has already been reported for volatile compounds in the juice of other fruit species, such as peach and apple, as well as in citrus [36,52,53]. In the study by Yu et al. [36], 25 QTLs were detected as stable over time, and among the same compounds also included in our study, 3 QTLs had close locations on the genetic map (same linkage groups). These are octanal, δ-cadinene and valencene, whose QTLs are positioned on linkage groups 8, 6 and 3, respectively. It would therefore appear that the same genes regulate the production of these volatile compounds in the peel but with tissue-specific expression of certain terpene synthases [54].

Some candidate genes involved in the biosynthesis of terpenoid compounds have been identified. Although the family of terpenes is large [6,8], only one terpene synthase was detected among the QTLs of scaffold 8, related to aliphatic aldehydes and monoterpenes. The geranylgeranyl pyrophosphate synthase is located on scaffold 5 and is associated with monoterpene QTLs. It is a key enzyme in the monoterpene and sesquiterpene pathways since it synthesizes the precursor of terpenes and terpenoids, the geranylgeranyl PP [7]. Further work is needed to verify whether this gene is the main player in the regulation of terpene synthesis, such as studying other progenies with different parents and, above all, offspring with more hybrids, to reduce the confidence interval of QTLs.

This is the first time that the inheritance of the PEO yield trait has been studied in citrus. Approximately half of the hybrids showed higher or lower oil yields than the two parents. This trait therefore has transgressive segregation and can be improved (almost doubled) by selecting hybrids from crosses between parents with a small difference in yield. Transgressive segregation is frequently observed for quantitative traits and represents an important component of adaptive evolution [55].

Marker-assisted selection (MAS) would be feasible for a number of compounds and for oil yield, with QTLs accounting for a significant portion of the total variance. Sesquiterpenes, such as δ-cadinene, valencene and α-sinensal, are compounds with weak aromatic properties and are therefore of little interest for breeding [56,57,58]. Aliphatic aldehydes (octanal, decanal, and dodecanal), on the other hand, appear to be a prime target for SAM as they are known to play a major role in orange aroma [25,56,58,59,60,61]. However, the complexity of an aromatic profile based on numerous compounds, some of which are at very low concentrations and sometimes undetectable, cannot be reproduced because most of the QTLs of these compounds are not detectable. Previous studies also revealed the complexity of the inheritance of sugar content and acidity, which are other key components of fruit quality [32,33,34,35]. QTLs vary among studies, as well as over the fruit-ripening period. This complexity of quality trait inheritance can explain why, compared with that in other crops, marker-assisted selection is still limited in citrus to a few traits with monogenic determinants, such as Alternaria brown spot resistance [62], Tristeza immunity of *Poncirus trifoliata* (L.) Raf., polyembryony or male sterility [63]. On the other hand, markers for QTLs of aromatic compounds can be used to vary the content of compounds and create new aroma profiles.

Ongoing extensive genomic and phenomic projects, genome-wide association studies and genomic selection approaches should improve our capacity to optimize the integration of omics for successful citrus-breeding programs.

## 5. Conclusions

Due to the heterozygosity of the parents, the composition of essential oils in the fruit peel of clementine × orange hybrids shows variations among the main class of components, but the high dominance of limonene masks this variation. However, some aroma compounds are present in very different proportions compared to those in the parents. Nevertheless, the yield of essential oils varies greatly between hybrids with often-positive transgressive values. The production of the main volatile compounds in the oil glands of the peel appears to be regulated by a small number of loci, mainly on chromosomes 5 and 8. This is particularly evident for monoterpenes and aliphatic aldehydes, whose QTLs colocalize to the same linkage groups. For sesquiterpenes, the QTLs are more widely distributed in the genome. Unfortunately, the aromatic descriptors ‘orange’ and ‘clementine’ could not be correlated with the number of volatile compounds. Marker-assisted selection seems to be a promising way to improve oil yield and to select hybrids containing high levels of aliphatic aldehydes, which are major molecules in the orange aroma profile.

## Figures and Tables

**Table 1:** Description of linkage groups (LGs): number of markers, size and marker density.

LG	Markers	Size (cM)	Mean Distance between SNPs (cM)	Max Distance between 2 SNP (cM)s
1	99	121	1.3	10.3
2	98	187	2.0	32.1
3	132	131	1.0	4.4
4	32	110	3.6	12.8
5	69	62	0.9	5.1
6	127	132	1.0	11.9
7	60	76	1.3	5.1
8	63	103	1.7	8.6
9	30	132	4.5	19.1
Total	710	1054	-	-

**Table 2:** Description of QTLs identified in the clementine–orange hybrid population.

Compound	LG ^1^	QTL Map Position ^2^	Physical Position ^3^	Max LOD ^4^	LOD Threshold ^5^	Var (%) ^6^	K-W ^7^
α-pinene	5	5–21–27	scaff_5_1891859–scaff_5_7341652–scaff_5_8671671	4.3	3.5	25.8	
	8	67–67–73	scaff_8_19483932–scaff_8_19483932–scaff_8_18742028	4.3	3.6	25.6	*****
sabinene	8	65–67–67	scaff_8_17641584–scaff_8_19483932–scaff_8_19483932	5.4	4.4	31.0	*******
β-pinene	8	65–67–67	scaff_8_17641584–scaff_8_19483932–scaff_8_19483932	4.8	4.1	28.0	******
octanal	8	46–75–77	scaff_8_2499192–scaff_8_19803231–scaff_8_20270007	5.6	4.3	31.9	*******
limonene	9	89–90–95	scaff_9_24887259–scaff_9_24887259–scaff_9_27099604	4.8	3.3	27.9	*******
	8	65–67–67	scaff_8_17641584–scaff_8_19483932–scaff_8_19483932	3.7	3.7	22.5	*****
citronellal	5	21–37–37	scaff_5_7341652–scaff_5_24327337–scaff_5_24327337	5.9	3.4	33.2	******
decanal	8	46–77–78	scaff_8_2499192–scaff_8_20270007–scaff_8_20292393	8.9	4.0	45.8	*******
neral	5	28–30–30	scaff_5_11337482–scaff_5_12169332–scaff_5_12169332	3.8	3.7	22.8	**
geranial	5	28–30–42	scaff_5_11337482–scaff_5_12169332–scaff_5_27088110	5.3	3.7	30.7	******
limonene-10-ol	8	48–52–77	scaff_8_2628498–scaff_8_4208594–scaff_8_20270007	11.6	4.7	55.0	*******
dodecanal	8	48–77–78	scaff_8_2359199–scaff_8_20270007–scaff_8_20292393	6.7	4.0	37.1	*******
valencene	3	96–104–104	scaff_3_19904227–scaff_3_21279770–scaff_3_21279770	5.6	4.7	32.0	***
α-farnesene	6	34–51–51	scaff_6_15868752–scaff_6_17711788–scaff_6_17893337	3.8	3.6	23.0	******
δ-cadinene	1	63–79–79	scaff_1_10400304–scaff_1_20460221–scaff_1_219122840	5.9	3.5	33.5	*******
	6	66–73–73	scaff_6_19700867–scaff_6_20697959–scaff_6_20697959	4.3	3.4	25.4	*****
α-sinensal	8	34–63–64	scaff_8_2413159–scaff_8_13317923–scaff_8_17514156	5.6	3.7	33.3	*******
PEO yield	5	3–8–21	scaff_5_1134031–scaff_5_2137822–scaff_5_3423139	4.9	3.4	28.7	******
	8	0–9–13	scaff_8_68590–scaff_8_33631–scaff_8_341083	3.3	3.0	20.5	******

^1^ Linkage groups: here each linkage group is associated with the scaffold corresponding to the physical map of the clementine tree. ^2^ Genetic position: the first value describes the first genetic position above the significance threshold, the second describes the max LOD position and the third describes the last position above the significance threshold. ^3^ Physical positions of the markers corresponding to the genetic positions of the QTLs. ^4^ LOD maximum score calculated for the QTL. ^5^ LOD score at which a significant difference is detected by permutation test (*p* ≤ 0.05). ^6^ Percentage of total variance explained by the QTL. ^7^ Significance level in the Kruskal–Wallis test. - *p* > 0.05; ** *p* < 0.01; *** *p* < 0.0001; ***** *p* < 0.00001; ****** *p* < 0.000001; ******* *p* < 0.0000001.

**Table 3:** Identified genes in aromatic compound QTLs related to terpenoid biosynthesis.

QTL	Scaf.	Gene	Start	End	Protein Name	Pathway
δ-cadinene	1	*CICLE_v10008448mg*	16657241	16660189	tyrosine transaminase family protein	Ubiquinone and other terpenoid–quinone biosynthesis
δ-cadinene	1	*CICLE_v10008222mg*	15860208	15866603	tRNA dimethylallyltransferase	Zeatin biosynthesis
δ-cadinene	6	*CICLE_v10012247mg*	20655671	20659398	preny protein peptidase	Terpenoid backbone biosynthesis
α-pinene	5	*CICLE_v10000884mg*	4487163	4490184	GA requiring 3	Isoprenoid biosynthetic process
		*CICLE_v10000902mg*	4498792	4501743		
		*CICLE_V10001179MG*	4550039	4553132		
α-pinene, citronellal	5	*CICLE_v10001398mg*	9330188	9333235	geranylgeranyl transferase	Ubiquinone and other terpenoid–quinone biosynthesis
	5	*CICLE_v10003969mg*	8303490	8304788	geranylgeranyl pyrophosphate synthase	Terpenoid backbone biosynthesis
		*CICLE_v10002201mg*	8359634	8360521		
		*CICLE_v10002259mg*	8409347	8410432		
α-pinene, citronellal, geranial	5	*CICLE_v10000921mg*	15445701	15450041	trans-cinnamate 4-monooxygenase	Ubiquinone and other terpenoid–quinone biosynthesis
	5	*CICLE_v10000962mg*	16114444	16118985	prenylcysteine oxidase/farnesylcysteine lyase	Terpenoid backbone biosynthesis
geranial	5	*CICLE_v10000735mg*	25965963	25969199	4-coumarate--CoA ligase	Ubiquinone and other terpenoid–quinone biosynthesis
	5	*CICLE_v10000923mg*	26496115	26502834	phosphomevalonate kinase	Terpenoid backbone biosynthesis (mevalonic pathway)
α-farnesene	6	*CICLE_v10012198mg*	16230126	16231785	undecaprenyl pyrophosphate synthetase family protein	Terpenoid backbone biosynthesis
dodecanal, octanal, decanal, limonene-10-ol = (dodl)	8	*CICLE_v10028350mg*	4340444	4344926	4-hydroxy-3-methylbut-2-enyl diphosphate reductase	Terpenoid backbone biosynthesis (non-mevaloic pathway)
	8	*CICLE_v10028537mg*	6315013	6322458	farnesyl-diphosphate farnesyltransferase	Sesquiterpenoid and triterpenoid biosynthesis
	8	*CICLE_v10029717mg*	15186679	15187280	NADH-Ubiquinone/plastoquinone (complex I) protein	Ubiquinone and other terpenoid–quinone biosynthesis
(dodl), sabinene, limonene, β-pinene	8	*CICLE_v10030056mg*	17909793	17910005	terpene synthase 14	Terpenoid backbone biosynthesis
α-sinensal	9	*CICLE_v10005360mg*	13264152	13266718	γ-tocopherol methyltransferase	Ubiquinone and other terpenoid–quinone biosynthesis

## Data Availability

The Illumina Hiseq4000 sequencing raw data are available in the NCBI SRA (Sequence Read Archive), under the BioProject number PRJNA986089.

## Supplementary Materials

The following supporting information can be downloaded at: https://www.mdpi.com/article/10.3390/genes14091800/s1, Table S1: PEO composition of the parent and the hybrids (each aromatic compounds was expressed in relative proportion); Table S2: Genes in QTL: position (in the reference genome), identity (GO) and corresponding Proteins (EMBL database); Figure S1: PCA biplots of the chemical diversity of the progeny and parents based on the 31 major aromatic compounds.

## References

[1] Teigiserova D.A., Tiruta-Barna L., Ahmadi A., Hamelin L., Thomsen M.A. (2021). Step Closer to Circular Bioeconomy for Citrus Peel Waste: A Review of Yields and Technologies for Sustainable Management of Essential Oils. J. Environ. Manag..

[2] Ferrer V., Paymal N., Costantino G., Paoli M., Quinton C., Tomi F., Luro F. (2023). Correspondence between the compositional and aromatic diversity of leaf and fruit essential oils and pomological diversity of 43 sweet oranges (*Citrus x aurantium* var *sinensis* L.). Plants.

[3] González-Mas M.C., Rambla J.L., López-Gresa M.P., Amparo Blázquez M., Granell A. (2019). Volatile Compounds in Citrus Essential Oils: A Comprehensive Review. Front. Plant Sci..

[4] Gershenzon J., Dudareva N. (2007). The Function of Terpene Natural Products in the Natural World. Nat. Chem. Biol..

[5] Tholl D. (2006). Terpene Synthases and the Regulation, Diversity and Biological Roles of Terpene Metabolism. Curr. Opin. Plant Biol..

[6] Degenhardt J., Köllner T.G., Gershenzon J. (2009). Monoterpene and Sesquiterpene Synthases and the Origin of Terpene Skeletal Diversity in Plants. Phytochemistry.

[7] Dudareva N., Klempien A., Muhlemann J.K., Kaplan I. (2013). Biosynthesis, Function and Metabolic Engineering of Plant Volatile Organic Compounds. New Phytol..

[8] Bohlmann J., Meyer-Gauen G., Croteau R. (1998). Plant Terpenoid Synthases: Molecular Biology and Phylogenetic Analysis. Proc. Natl. Acad. Sci. USA.

[9] Maruyama T., Ito M., Honda G. (2001). Molecular Cloning, Functional Expression and Characterization of (E)-b-Farnesene Synthase from *Citrus junos*. Biol. Pharm. Bull..

[10] Lücker J., El Tamer M.K., Schwab W., Verstappen F.W.A., Van der Plas L.H.W., Bouwmeester H.J., Verhoeven H.A. (2002). Monoterpene Biosynthesis in Lemon (*Citrus limon*): CDNA Isolation and Functional Analysis of Four Monoterpene Synthases. Eur. J. Biochem..

[11] Sharon-Asa L., Shalit M., Frydman A., Bar E., Hollandetti O.D., Lavi U., Lewinsohn E., Eyal Y. (2003). Citrus Fruit Flavor and Aroma Biosynthesis: Isolation, Functional Characterization, and Developmental Regulation of Cstps1, a Key Gene in the Production of the Sesquiterpene Aroma Compound Valencene: Characterization and Regulation of Valencene Synthase. Plant J..

[12] Shimada T., Endo T., Fujii H., Omura M. (2005). Isolation and Characterization of a New D-Limonene Synthase Gene with a Different Expression Pattern in *Citrus unshiu* Marc. Sci. Hortic..

[13] Shimada T., Endo T., Fujii H., Hara M., Ueda T., Kita M., Omura M. (2004). Molecular Cloning and Functional Characterization of Four Monoterpene Synthase Genes from *Citrus unshiu* Marc. Plant Sci..

[14] Dornelas M.C., Mazzafera P.A. (2007). Genomic Approach to Characterization of the Citrus Terpene Synthase Gene Family. Genet. Mol. Biol..

[15] Kohzaki K., Gomi K., Yamasaki-Kokudo Y., Ozawa R., Takabayashi J., Akimitsu K. (2009). Characterization of a Sabinene Synthase Gene from Rough Lemon (*Citrus jambhiri*). J. Plant Physiol..

[16] Rodríguez A., San Andrés V., Cervera M., Redondo A., Alquézar B., Shimada T., Gadea J., Rodrigo M., Zacarías L., Palou L. (2011). The Monoterpene Limonene in Orange Peels Attracts Pests and Microorganisms. Plant Signal. Behav..

[17] Rodríguez A., Shimada T., Cervera M., Redondo A., Alquézar B., Rodrigo M.J., Zacarías L., Palou L., López M.M., Peña L. (2015). Resistance to Pathogens in Terpene Down-Regulated Orange Fruits Inversely Correlates with the Accumulation of D-Limonene in Peel Oil Glands. Plant Signal. Behav..

[18] Rodríguez A., Peris J.E., Redondo A., Shimada T., Costell E., Carbonell I., Rojas C., Peña L. (2017). Impact of D-Limonene Synthase up- or down-Regulation on Sweet Orange Fruit and Juice Odor Perception. Food Chem..

[19] Wu G.A., Prochnik S., Jenkins J., Salse J., Hellsten U., Murat F., Perrier X., Ruiz M., Scalabrin S., Terol J. (2014). Sequencing of Diverse Mandarin, Pummelo and Orange Genomes Reveals Complex History of Admixture during Citrus Domestication. Nat. Biotechnol..

[20] Xu Q., Chen L.-L., Ruan X., Chen D., Zhu A., Chen C., Bertrand D., Jiao W.-B., Hao B.-H., Lyon M.P. (2013). The Draft Genome of Sweet Orange (*Citrus sinensis*). Nat. Genet..

[21] Luro F., Curk F., Froelicher Y., Ollitrault P., Zech-Matterne V., Girolamo F. (2017). Recent Insights on Citrus Diversity and Phylogeny. AGRUMED: Archaeology and History of Citrus Fruit in the Mediterranean: Acclimatization, Diversifications, Uses.

[22] Alquézar B., Rodríguez A., de la Peña M., Peña L. (2017). Genomic Analysis of Terpene Synthase Family and Functional Characterization of Seven Sesquiterpene Synthases from *Citrus sinensis*. Front. Plant Sci..

[23] Kerbiriou P., Plotto A., Goodner K., Baldwin E., Gmitter F.G. (2007). Distribution of Aroma Volatiles in a Population of Tangerine Hybrids. Proc. Fla. State Hort. Soc..

[24] Barboni T., Luro F., Chiaramonti N., Desjobert J.-M., Muselli A., Costa J. (2009). Volatile Composition of Hybrids Citrus Juices by Headspace Solid Phase Micro Extraction/Gas Chromatography/Mass Spectrometry. Food Chem..

[25] Miyazaki T., Plotto A., Goodner K., Gmitter F.G. (2011). Distribution of Aroma Volatile Compounds in Tangerine Hybrids and Proposed Inheritance. J. Sci. Food Agric..

[26] Ruberto G., Biondi D., Piattelli M., Rapisarda P., Starrantino A. (1993). Profiles of Essential Oils of New Citrus Hybrids. Flavour Fragr. J..

[27] Ruberto G., Rapisarda P. (2002). Essential Oils of New Pigmented Citrus Hybrids: *Citrus sinensis* L. Osbeck x *C clementina* Hort. Ex Tanaka. J. Food Sci..

[28] Verzera A., Trozzi A., Zappalá M., Condurso C., Cotroneo A. (2005). Essential Oil Composition of *Citrus meyerii* Y. Tan. and *Citrus medica* L. Cv. Diamante and Their Lemon Hybrids. J. Agric. Food Chem..

[29] Verzera A., Tripodi G., Cotroneo A. (2009). Characteristics of a New Citrus Hybrid Essential Oil, *Citrus clementina* Cv. Nules x *Citrus limon* Cv. Cavone. J. Essent. Oil Bear. Plants.

[30] Fabroni S., Ruberto G., Rapisarda P. (2012). Essential Oil Profiles of New Citrus Hybrids, a Tool for Genetic Citrus Improvement. J. Essent. Oil Res..

[31] Tomi F., Barzalona M., Casanova J., Luro F. (2008). Chemical Variability of the Leaf Oil of 113 Hybrids from *Citrus clementina* (Commun) × *Citrus deliciosa* (Willow Leaf). Flavour Fragr. J..

[32] Yu Y., Chen C., Gmitter F.G. (2016). QTL Mapping of Mandarin (*Citrus reticulata*) Fruit Characters Using High-Throughput SNP Markers. Tree Genet. Genomes.

[33] Imai A., Yoshioka T., Hayashi T. (2017). Quantitative Trait Locus (QTL) Analysis of Fruit Quality Traits for Mandarin Breeding in Japan. Tree Genet. Genomes.

[34] Curtolo M., Cristofani-Yaly M., Gazaffi R., Takita M.A., Figueira A., Machado M.A. (2017). QTL Mapping for Fruit Quality in Citrus Using DArTseq Markers. BMC Genom..

[35] Khefifi H., Dumont D., Costantino G., Doligez A., Anna Carla B., Bérard A., Morillon R., Ollitrault P., Luro F. (2022). Mapping of QTLs for citrus quality traits throughout the fruit maturation process on clementine (*Citrus reticulata* x *C. sinensis*) and mandarin (*C. reticulata* Blanco) genetic maps. TGG.

[36] Yu Y., Bai J., Chen C., Plotto A., Yu Q., Baldwin E.A., Gmitter F.G. (2017). Identification of QTLs Controlling Aroma Volatiles Using a ‘Fortune’ x ‘Murcott’ (*Citrus reticulata*) Population. BMC Genom..

[37] Shimada T., Endo T., Fujii H., Rodríguez A., Yoshioka T., Peña L., Omura M. (2021). Biological and Molecular Characterization of Linalool-Mediated Field Resistance against *Xanthomonas citri* Subsp. *citri* in Citrus Trees. Tree Physiol..

[38] Butelli E., Licciardello C., Zhang Y., Liu J., Mackay S., Bailey P., Reforgiato-Recupero G., Martin C. (2012). Retrotransposons Control Fruit-Specific, Cold-Dependent Accumulation of Anthocyanins in Blood Oranges. Plant Cell.

[39] Luro F., Bloquel E., Tomu B., Costantino G., Tur I., Riolacci S., Varamo F., Ollitrault P., Froelicher Y., Curk F., Zech-Matterne V., Girolamo F. (2017). The INRA-CIRAD citrus germplasm collection of San Giuliano, Corsica. AGRUMED: Archaeology and History of Citrus Fruit in the Mediterranean: Acclimatization, Diversifications, Uses.

[40] Bicchi C., Liberto E., Matteodo M., Sgorbini B., Mondello L., d’Acampora Zellner B., Costa R., Rubiolo P. (2008). Quantitative Analysis of Essential Oils: A Complex Task. Flavour Fragr. J..

[41] Oueslati A., Salhi-Hannachi A., Luro F., Vignes H., Mournet P., Ollitrault P. (2017). Genotyping by Sequencing Reveals the Interspecific *C. maxima*/*C. reticulata* Admixture along the Genomes of Modern Citrus Varieties of Mandarins, Tangors, Tangelos, Orangelos and Grapefruits. PLoS ONE.

[42] Sonah H., Bastien M., Iquira E., Tardivel A., Légaré G., Boyle B., Normandeau E., Laroche J., LaRose S., Jean M. (2013). An Improved Genotyping by Sequencing (GBS) Approach Offering Increased Versatility and Efficiency of SNP Discovery and Genotyping. PLoS ONE.

[43] Turner S.D. (2018). qqman: An R package for visualizing GWAS results using Q-Q and manhattan plots. J. Open Source Softw..

[44] Luro F., Baccati C., Paoli M., Marchi E., Costantino G., Gibernau M., Ollitrault P., Tomi F. (2022). Phylogenetic and taxonomic status of *Citrus halimii* B.C. Stone determined by genotyping complemented by chemical analysis of leaf and fruit rind essential oils. Sci. Hortic..

[45] Lota M.-L., de Rocca Serra D. (1999). Chemical Composition of Peel and Leaf Essential Oils of *Citrus medica* L. and *C. limonimedica* Lush. Flavour Fragr. J..

[46] Lota M.-L., de Rocca Serra D., Tomi F., Casanova J. (2000). Chemical Variability of Peel and Leaf Essential Oils of Mandarins from *Citrus reticulata* Blanco. Biochem. Syst. Ecol..

[47] Lota M.-L., de Rocca Serra D., Tomi F., Jacquemond C., Casanova J. (2002). Volatile Components of Peel and Leaf Oils of Lemon and Lime Species. J. Agric. Food Chem..

[48] Ferrer V., Paymal N., Quinton C., Tomi F., Luro F. (2022). Investigations of the Chemical Composition and Aromatic Properties of Peel Essential Oils throughout the Complete Phase of Fruit Development for Two Cultivars of Sweet Orange (*Citrus sinensis* (L.) Osb.). Plants.

[49] Paun O., Fay M.F., Soltis D.E., Chase M.W. (2007). Genetic and Epigenetic Alterations after Hybridization and Genome Doubling. TAXON.

[50] Miyazaki T., Plotto A., Baldwin E.A., Reyes-De-Corcuera J.I., Gmitter F.G. (2012). Aroma Characterization of Tangerine Hybrids by Gas-Chromatography Olfactometry and Sensory Evaluation. J. Sci. Food Agric..

[51] Perez-Cacho P.R., Rouseff R.L. (2008). Fresh Squeezed Orange Juice Odor: A Review. Food Sci. Nutr..

[52] Eduardo I., Chietera G., Pirona R., Pacheco I., Troggio M., Banchi E., Bassi D., Rossini L., Vecchietti A., Pozzi C. (2013). Genetic Dissection of Aroma Volatile Compounds from the Essential Oil of Peach Fruit: QTL Analysis and Identification of Candidate Genes Using Dense SNP Maps. Tree Genet. Genomes.

[53] Souleyre E.J.F., Chagné D., Chen X., Tomes S., Turner R.M., Wang M.Y., Maddumage R., Hunt M.B., Winz R.A., Wiedow C. (2014). The AAT1 Locus Is Critical for the Biosynthesis of Esters Contributing to ‘Ripe Apple’ Flavour in ‘Royal Gala’ and ‘Granny Smith’ Apples. Plant J..

[54] Shimada T., Endo T., Fujii H., Hara M., Omura M. (2005). Isolation and Characterization of (E)-Beta-Ocimene and 1,8 Cineole Synthases in *Citrus unshiu* Marc. Plant Sci..

[55] De los Reyes B.G. (2019). Genomic and Epigenomic Bases of Transgressive Segregation—New Breeding Paradigm for Novel Plant Phenotypes. Plant Sci..

[56] Högnadóttir Á., Rouseff R.L. (2003). Identification of Aroma Active Compounds in Orange Essence Oil Using Gas Chromatography–Olfactometry and Gas Chromatography–Mass Spectrometry. J. Chromatogr..

[57] Elston A., Lin J., Rouseff R. (2005). Determination of the Role of Valencene in Orange Oil as a Direct Contributor to Aroma Quality. Flavour Fragr. J..

[58] Qiao Y., Xie B., Zhang Y., Zhang Y., Fan G., Yao X., Pan S. (2008). Characterization of Aroma Active Compounds in Fruit Juice and Peel Oil of Jinchen Sweet Orange Fruit (*Citrus sinensis* (L.) Osbeck) by GC-MS and GC-O. Molecules.

[59] Gaffney B.M., Havekotte M., Jacobs B., Costa L., Ho C.-T.H. (1996). CharmAnalysis of Two *Citrus sinensis* Peel Oil Volatiles. Perfum. Flavorist.

[60] Buettner A., Mestres M., Fischer A., Guasch J., Schieberle P. (2003). Evaluation of the Most Odour-Active Compounds in the Peel Oil of Clementines (*Citrus reticulata* Blanco Cv. Clementine). Eur. Food Res. Technol..

[61] Deterre S., Leclair C., Bai J., Baldwin E.A., Narciso J.A., Plotto A. (2016). Chemical and Sensory Characterization of Orange (*Citrus sinensis*) Pulp, a by Product of Orange Juice Processing Using Gas-Chromatography-Olfactometry. J. Food Qual..

[62] Cuenca J., Aleza P., Garcia-Lor A., Ollitrault P., Navarro L. (2016). Fine mapping for identification of citrus alternaria brown spot candidate resistance genes and development of new SNP markers for marker-assisted selection. Front. Plant Sci..

[63] Montalt R., Cuenca J., Vives M.C., Mournet P., Navarro L., Ollitrault P., Aleza P. (2023). Genotyping by sequencing for SNP-Based linkage analysis and the development of KASPar markers for male sterility and polyembryony in citrus. Plants Adv. Breed. Genet. Genom. Citrus.

