# Supplementary material for: Inheritance and Quantitative Trait Loci Mapping of Aromatic Compounds from Clementine (Citrus × clementina Hort. ex Tan.) and Sweet Orange (C. × sinensis (L.) Osb.) Fruit Essential Oils
Source: Genes (Basel). 2023 Sep 14;14(9):1800. doi: 10.3390/genes14091800 (PMC10531275; doi:10.3390/genes14091800)
Supplement: Supplementary file 1 [file genes-14-01800-s001.zip › SupplementaryData/Supplementary figure S1.pdf]

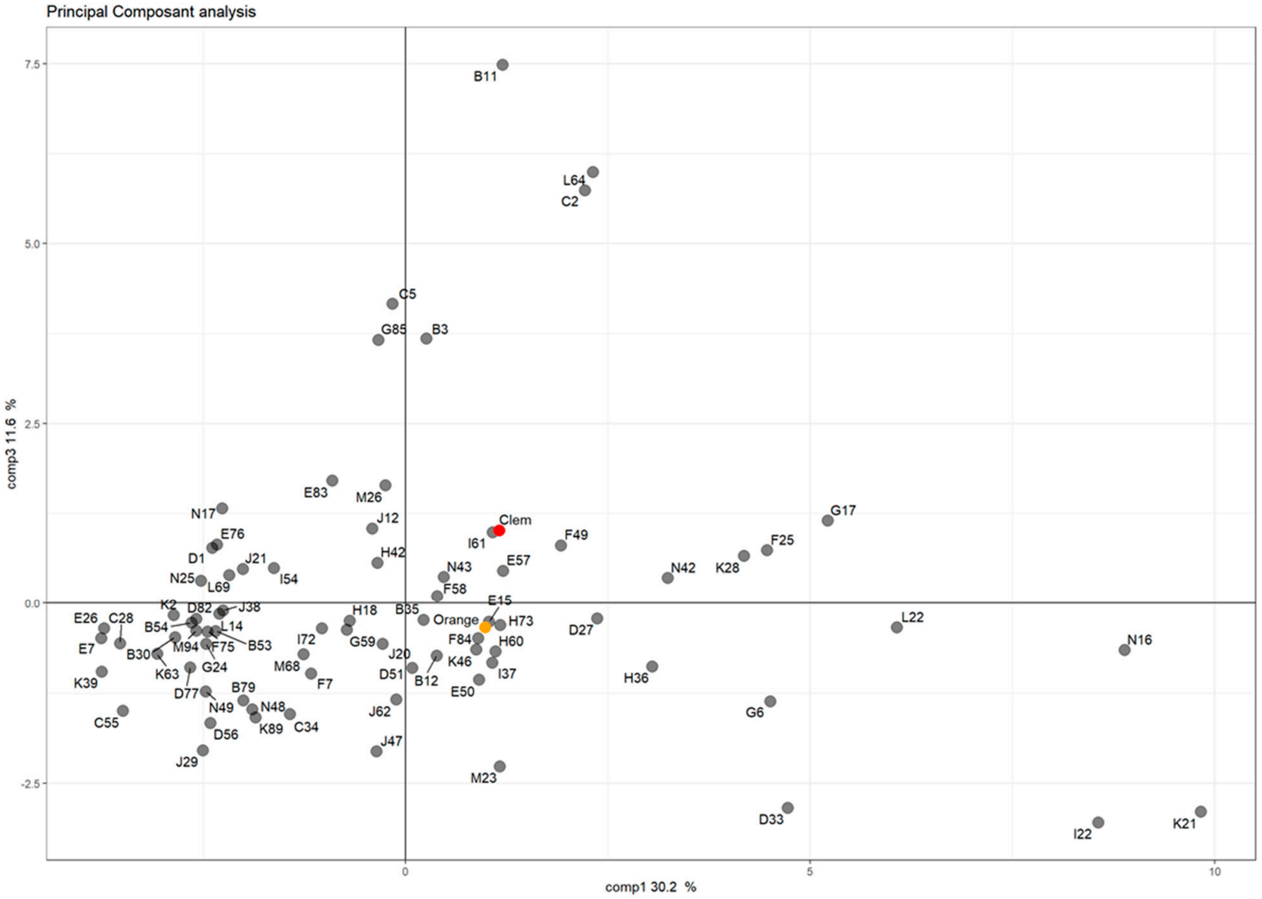

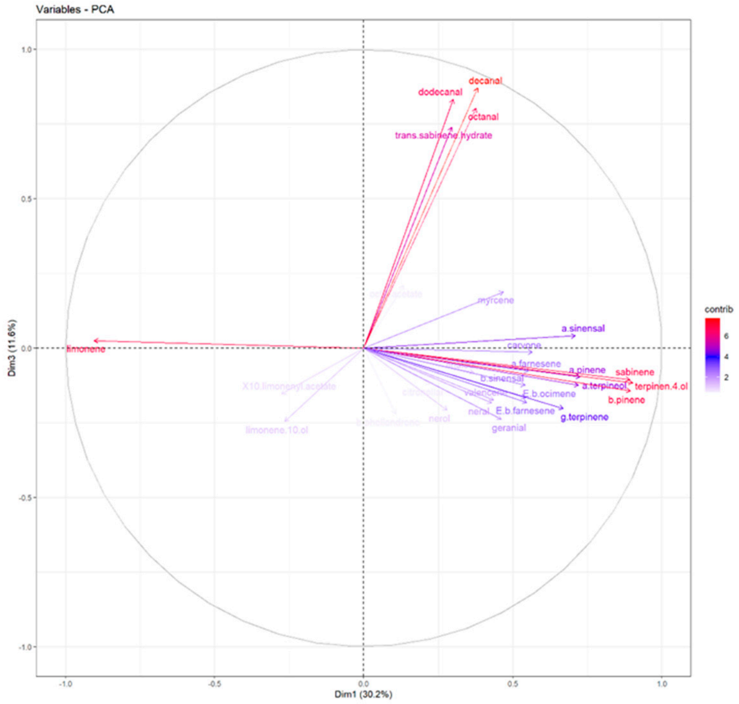

Supplementary figure S1: PCA representing the diversity of the progeny and its parents based only on the compounds with higher proportion than 0.1% (up figure) and the contribution of the compounds to this dispersion (low figure)
